# The effect of perioperative blood transfusion on kidney functions in total knee Arthroplasty

**DOI:** 10.12669/pjms.35.3.259

**Published:** 2019

**Authors:** Baran Sen, Ramadan Ozmanevra

**Affiliations:** 1 *Dr. Baran Sen, Department of Orthopaedics and Trauma Surgery, Cesme State Hospital, Izmir, Turkey*; 2 *Dr. Ramadan Ozmanevra, Department of Orthopaedics and Trauma Surgery, University of Kyrenia, Kyrenia, Northern Cyprus*

**Keywords:** Blood transfusion, Blood urea nitrogen, Glomerular filtration, Kidney function, Total knee arthroplasty

## Abstract

**Objective:**

Various studies have examined the effects of perioperative blood transfusion on kidney functions. In this study, we investigated the effects of blood transfusion on the kidney functions of patients undergoing total knee arthroplasty.

**Methods:**

: This retrospective study was carried out on 216 patients who had undergone total knee arthroplasty between January 2016 and January 2017. The patients were divided into two groups according to the level of blood transfusion used during surgery. Group-1 comprised 157 patients (72.7%) needing a blood transfusion of less than three units, while the 59 (27.3) patients in Group-2 required three or more than three units.

**Results:**

No statistical difference was found between the two groups regarding hypertension before surgery, diabetes mellitus, chronic kidney failure, smoking habits or lung disease (p> 0.05). Likewise, there was no significant difference related to pulmonary and other complications or mortality (p> 0.05). When the groups were compared according to their blood parameters, no statistical differences in postoperative renal or other system functions were found (p> 0.05).

**Conclusions:**

Blood transfusion does not have a negative effect on postoperative blood urea nitrogen (BUN) and creatinine levels, or glomerular filtration rate in total knee arthroplasty.

## INTRODUCTION

In USA 3.9% of all blood transfusions used during orthopedic surgery are during knee arthroplasty.^1^,^2^ This increase in perioperative blood transfusions has resulted in a rise in mortality and morbidity rates.^1^,^3^

Complications that may develop perioperatively in patients having blood transfusions include hemolytic and allergic reactions during surgery, transfusion-associated acute lung damage, transfusion-associated circulatory overload, graft versus host disease and infection.^4^,^5^ A further complication of perioperative blood transfusions mentioned in many studies, is kidney dysfunction.^6^-^8^

During our literature search, we found many studies looking at the effects of perioperative blood transfusions. However, there were no studies in the field of orthopedic surgery that specifically examined these effects during total knee prosthesis surgery. Therefore, our study focuses on the effects of perioperative blood transfusion on the kidney functions of patients undergoing total knee arthroplasty.

## METHODS

A series of 216 patients who had undergone total knee arthroplasty between January 2016 and January 2017 was reviewed retrospectively. For this purpose, ethical approval was obtained (35/2018, Bozyaka Education and Research Hospital Clinical Research Ethics Committee).

Patients’ age, gender and smoking habits; any history of hypertension, diabetes mellitus, chronic obstructive lung disease and chronic kidney disease were noted. Duration of hospital stay, the type of anesthesia used, preoperative and postoperative blood glucose levels, perioperative blood transfusion amounts, postoperative pulmonary complications, other postoperative complications and postoperative morbidity and mortality were also recorded, as well as preoperative and postoperative urea, creatinine, aspartate aminotransferase(AST), alanine aminotransferase (ALT) and glomerular filtration rates. Glomerular filtration rates were calculated using MDRD formula (Modification of Diet in Renal Diseases Study).

One hundred and fifty-seven patients (72,7%) who received a blood transfusion of less than three units were categorized as Group-1; 59 (27.3%)patients receiving 3 or more units comprised Group-2. Comparison of the two groups focused primarily on preoperative and postoperative renal functions. Hematocrit (hct) levels of patients receiving a transfusion were under 24% and hemodialysis was not required for any of the patients. Any patients with a preoperative creatinine level of 2mg /dl or over were excluded from the study.

Data analysis was carried out using SPSS 15.0 for Windows software with 95% confidence interval. Continuous variables were categorized and compared according to chi-square test and fisher’s exact chi square test. T-test was used for group comparison of variables. The level of significance was set at p-value less than 0.05.

## RESULTS

No difference was found between the preoperative data of both groups regarding age, gender, hypertension, diabetes mellitus, chronic lung disease, chronic kidney failure or smoking habit (p> 0.05) (Table-I). The preoperative urea, creatinine, glomerular filtration rate (GFR), aspartate aminotransferase (AST) and alanine aminotransferase (ALT) levels were statistically similar(p>0.05) (Table-I).

**Table-I:** Preoperative patient data.

Preoperative Patient	Group-1 (n:157)	Group-2 n:59	p
Age	65± 8.114	66±8.239	0,524
Gender (Female)	133(84.7%)	52(88.1%)	0.523
Gender (Male)	24(15.3%)	7(11.9%)	0.523
Hypertension presence	86(54.8%)	35(59.3%)	0.549
Diabetes Mellitus presence	61(38.9%)	21(35.6%)	0.660
Chronic Obstructive Lung Disease	26(16.6%)	7(11.9%)	0.393
Chronic Kidney Failure	5(3.2%)	1(1.7%)	0.553
Smoking	36(22.9%)	10 (16.9%)	0.339
BUN	37.5±12.292	38.4±11.089	0.624
Creatinine	0.83±0.0203	0.83±0.198	0.983
AST	20.9±6.456	20.9±6.380	0.553
ALT	18.4±7.630	19±8.024	0.999
GFR	61.3±4.896	60.9±5.607	0.580

When length of postoperative hospital stay was compared between the two groups, the duration was seen to be longer in Group-2 (p<0.05). Since most patients had elective surgery, spinal anesthesia was used most frequently (92.1%). There was no significant difference between the groups regarding the anesthesia type applied (p>0.05) (Table-II).

**Table-II:** Perioperative patient data.

Perioperative Clinical Data	Group-1 (n:157)	Group-2 (n:59)	p
General anesthesia	11 (7%)	6 (10.2%)	0.442
Spinal anesthesia	146 (93%)	53 (89.8%)	0.442
Mortality	0	0	-
Postoperative pulmonary complication	0	0	-
Other postoperative complications	0	0	-
Duration of hospital stay	6±0.8	6.3±0.87	0.016

The groups did not differ significantly with regard to preoperative and postoperative pulmonary complications or mortality (p> 0.05) (Table-II). When the two groups were compared, no statistical difference was found regarding postoperative kidney or other system functions (p> 0.05) (Table-III).

**Table-III:** Postoperative blood values.

Postoperative Blood Values	Group-1 (n:157)	Group-2 (n:59)	p
BUN	30±9.990	32.7±10.653	0.086
Creatinine	0.72±0.165	0.72±0.204	0.827
AST	23.4±12.739	22.9±9.083	0.793
ALT	16.5±12.905	16.8±7.854	0.870
GFR	62.8±2.870	62.5±4.103	0.583

When blood parameter changes were reviewed between the groups, preoperatively and postoperatively, no significant difference was found statistically regarding levels of urea or creatinine; AST (aspartate aminotransferase); ALT (alanine aminotransferase); or glomerular filtration rates (GFR)(p> 0.05) (Table-IV).

**Table-IV:** Comparison of preoperative and postoperative blood values of Group 1 and 2.

Blood value	Group 1 n:157	p	Group 2 n:59	p
Preop BUN	37.56±12.292	<0.001	38.46±11.089	<0.001
Postop BUN	30.09±9.990		32.77±10.653	
Preop Creatinine	0.83±0.203	<0.001	0.83±0.198	<0.001
Postop Creatinine	0.72±0.165		0.72±0.204	
Preop GFR	61.39±4.896	<0.001	60.93±5.607	0.004
Postop GFR	62.81±2.870		62.54±0.534	
Preop AST	20.98±6.456	0.017	20.98±6.380	0.117
Postop AST	23.45±12.739		22.97±9.083	
Preop ALT	18.41±7.630	0.061	19.06±8.024	0.038
Postop ALT	16.57±12.905		16.86±7.854	

## DISCUSSION

As the number of orthopedic surgical procedures rises, there is a corresponding increase in the incidence of perioperative blood transfusions. Patients undergoing total knee arthroplasty frequently receive blood transfusions. Along with an escalation in the financial burden, many studies have reported blood transfusion-associated medical complications.^1^,^9^,^10^ Transfusion-associated postoperative complications such as hemolytic, allergic reactions, transfusion-associated acute lung injury, transfusion-associated circulatory overload, graft versus host disease and infection and death after blood transfusion have been noted in many orthopedic studies and postoperative studies in other fields.^2^,^5^,^11^

Whitlock EL et al. found that in a large retrospective cohort of patients undergoing a diverse variety of surgeries, perioperative transfusion of a single unit of red blood cells is associated with an increased risk of perioperative ischemic stroke or myocardial infarction.^12^ In contrast to this study, no cardiac complication was observed in our study. Some studies have found the relationship between transfusion and the risk of infection following total joint arthroplasty.^13^,^14^ Friedman R et al. investigated whether allogeneic blood transfusion increases the risk of postoperative infection compared with autologous blood transfusion or no transfusion. The rates of infection in patients receiving no transfusion or autologous blood transfusion were similar. They conclude that the rates of any infection, lower or upper respiratory tract and lung infection, and wound inflammation or infection were significantly increased after elective total hip or total knee arthroplasty in patients receiving allogeneic blood transfusion compared with those receiving autologous blood transfusion or no blood transfusion.^13^ In our study, we found no statistically significant difference between the groups regarding lung complication rates.

According to a study by Ponnusamy KE et al., the effects of blood transfusion in orthopedic surgery include allergic reactions (21%), and transfusion-associated acute lung injury (27%). Amongst these complications, the most common causes of mortality are graft versus host disease (85-100%), transfusion-associated circulatory overload (2-15%), and transfusion-associated acute lung injury (5-10%).^2^ Furthermore, other researchers have found that blood transfusions lead to an increased risk of viral transmission and immunosuppression.^8^-^10^,^15^

Our findings were not in consistent with the literature. We found no significant statistical difference between the groups regarding lung complication rates. Although very few in number, there were complications of a comparable nature such as minor allergic and hemolytic reactions which were not recorded in our database.

Some researchers have investigated the relationship between perioperative blood transfusion and delirium among older people undergoing elective orthopedic surgery. They found that blood transfusion during surgery was an independent risk factor for post-operative delirium.^16^

Another complication of perioperative blood transfusion is the postoperative deterioration in kidney function. Cardiovascular surgery reports are commonly referred to in orthopedic literature.^8^,^15^,^17^ In a study the effects of blood transfusion had investigated in coronary by-pass surgery. Kuduvalli M et al. compared transfused patients with non-transfused patients. They found statistically significant increase for renal failure in transfused group. When examining postoperative morbidity and mortality, the study found the rate of kidney dysfunction in coronary bypass patients to be 2.6%.^7^ Whitson BA et al. investigated complications after blood product transfusion. Acute renal failure was significantly difference in receiving blood product transfusion patients.^9^ In our study no significant difference was found statistically regarding levels of urea or creatinine or glomerular filtration rates.

Another study reviewed the correlation between the use of blood products and acute kidney failure. According to this study, the incidence of kidney failure in a group receiving transfusion was 8%, compared to 1.8% in the non-transfused group; this was considered statistically significant.^18^

Satoglu IS et al. compared two groups for blood parameters showing postoperative renal and other system functions and detected no statistical differences. In this study, we evaluated GFR for renal functions in addition to the other values. No statistically significant difference was found between the groups regarding kidney functions.^1^

Our study examines the effects of perioperative blood transfusion on kidney functions. In cardiovascular surgery literature, studies indicate that blood transfusions affect kidney functions adversely; however, there are very few studies focusing on this topic in the field of orthopedic surgery. Despite the understanding that kidney functions are affected in a negative way in cardiovascular surgery, our study showed no negative effect on kidney functions of transfusion during total knee arthroplasty.

### Limitations of the study

It is a retrospective study, and the patient population is small.

## CONCLUSION

There is no doubt that postoperative complication rates increase with perioperative blood transfusion. However, contrary to other surgical disciplines, our orthopedic surgery study did not show any negative effect of blood transfusions on kidney functions in total knee arthroplasty. In order to confirm these findings larger prospective patient series and more parameters are required.

### Author`s Contribution

**BS** conceived, designed and did statistical analysis & editing of manuscript.

**BS & RO** did data collection and manuscript writing.

**RO** did review and final approval of manuscript.
